# Quantifying salinity in calcareous soils through advanced spectroscopic models: A comparative study of random forests and regression techniques across diverse land use systems

**DOI:** 10.1371/journal.pone.0307853

**Published:** 2024-08-22

**Authors:** Mohammad Tahmoures, Afshin Honarbakhsh, Sayed Fakhreddin Afzali, Mehdi Nourzadeh Hadad, Yaser Ostovari

**Affiliations:** 1 Department of Soil Conservation and Watershed Management, Zanjan Agricultural and Natural Resources Research Center, AREEO, Zanjan, Iran; 2 Department of Nature Engineering, Faculty of Natural Resources and Earth Sciences, Shahrekord University, Shahrekord, Iran; 3 Department of Natural Resource and Environmental Engineering, School of Agriculture, Shiraz University, Shiraz, Iran; 4 Soil Science Department, Nuclear Agriculture Research School, Nuclear Science and Technology Research Institute, Tehran, Iran; 5 Department of Soil Science, School of Agriculture, Shiraz University, Shiraz, Iran; Universiti Kebangsaan Malaysia, MALAYSIA

## Abstract

Precise prediction of soil salinity using visible, and near-infrared (vis-NIR) spectroscopy is crucial for ensuring food security and effective environmental management. This paper focuses on the precise prediction of soil salinity utilizing visible and near-infrared (vis-NIR) spectroscopy, a critical factor for food security and effective environmental management. The objective is to utilize vis-NIR spectra alongside a multiple regression model (MLR) and a random forest (RF) modeling approach to predict soil salinity across various land use types, such as farmlands, bare lands, and rangelands accurately. To this end, we selected 150 sampling points representatives of these diverse land uses. At each point, we collected soil samples to measure the soil salinity (ECe) and employed a portable spectrometer to capture the spectral reflectance across the full wavelength range of 400 to 2400 nm. The methodology involved using both individual spectral reflectance values and combinations of reflectance values from different wavelengths as input variables for developing the MLR and RF models. The results indicated that the RF model (RMSE = 4.85 dS m^-1^, R^2^ = 0.87, and RPD = 3.15), utilizing combined factors as input variables, outperformed others. Furthermore, our analysis across different land uses revealed that models incorporating combined input variables yielded significantly better results, particularly for farmlands and rangelands. This study underscores the potential of combining vis-NIR spectroscopy with advanced modeling techniques to enhance the accuracy of soil salinity predictions, thereby supporting more informed agricultural and environmental management decisions.

## 1. Introduction

Salt-affected soils are prevalent worldwide, particularly in regions characterized by low precipitation, high evaporation rates, and elevated water tables [1, 2]. Soil salinization poses a significant environmental challenge, impacting agricultural activities, sustainable productivity, development, and water quality [3, 4]. The presence of high concentrations of water-soluble salts in the soil contributes to detrimental land degradation processes, such as soil structure loss, crust formation, and soil dispersion [5, 6]. Soil salinization is particularly prominent in semi-arid and arid regions due to limited rainfall [7]. Therefore, obtaining a comprehensive and real-time understanding of soil salinity, while maintaining low costs and high efficiency, becomes crucial for effectively managing soil salinization and facilitating land use planning, especially in semi-arid and arid regions [7].

Soil salinity refers to the concentration of salt in the soil, typically measured in dS m^-1^ through the application of a water solution in a laboratory setting [1].Conventional approaches to measure soil salinity involve time-consuming laboratory analysis and field-based aerial surveys, which can be particularly challenging when dealing with large areas [1]. However, different types of minerals present in the soil exhibit distinct spectral behaviors related to salt content. Consequently, non-destructive measurement techniques have gained momentum. Two valuable sources of data in this regard are visible and near-infrared (Vis-NIR) spectroscopy and remote sensing data.

Numerous scientific studies have underscored the considerable potential of remotely sensed data, particularly utilizing platforms like Sentinel-2 MSI and Landsat-8 OLI, for effectively predicting soil salinity across expansive spatial scales through repetitive measurements [8–12]. These advanced remote sensing technologies provide valuable insights into soil salinity dynamics, enabling researchers to monitor and assess the extent of salinization over time. However, in recent decades, researchers have increasingly explored the utilization of visible and near-infrared (Vis-NIR) spectra data, spanning a wavelength range of 350 to 2500 nm, combined with different types of models. This novel approach has demonstrated significant potential for accurately estimating soil salinity levels [13, 14]. For instance, a study was conducted using Vis-NIR spectra data to investigate the influence of soil salinity and moisture on spectral features. Their findings shed light on the spectral response patterns associated with varying levels of salinity and moisture content, providing crucial insights into the spectral behavior of salt-affected soils [13].

While previous studies have explored the potential of Vis-NIR spectra data in predicting soil salinity, a research gap remains regarding its application to calcareous soils and different types of land use. Existing studies have primarily focused on sampled points without considering the specific land use categories, limiting the generalizability of their findings. Therefore, there is a need to investigate the predictive capabilities of Vis-NIR spectra data for soil salinity under various land use types, particularly in calcareous soils, to improve model accuracy and applicability. The objective of this study was to develop a i) statistical model (i.e. MLR) and ii) machine learning model (specifically employing the random forest algorithm) in the central part of Iran using Vis-NIR spectra data at whole study area and different types of land use.

## 2. Material and methods

### 2.1. Study area

**Fig 1:**
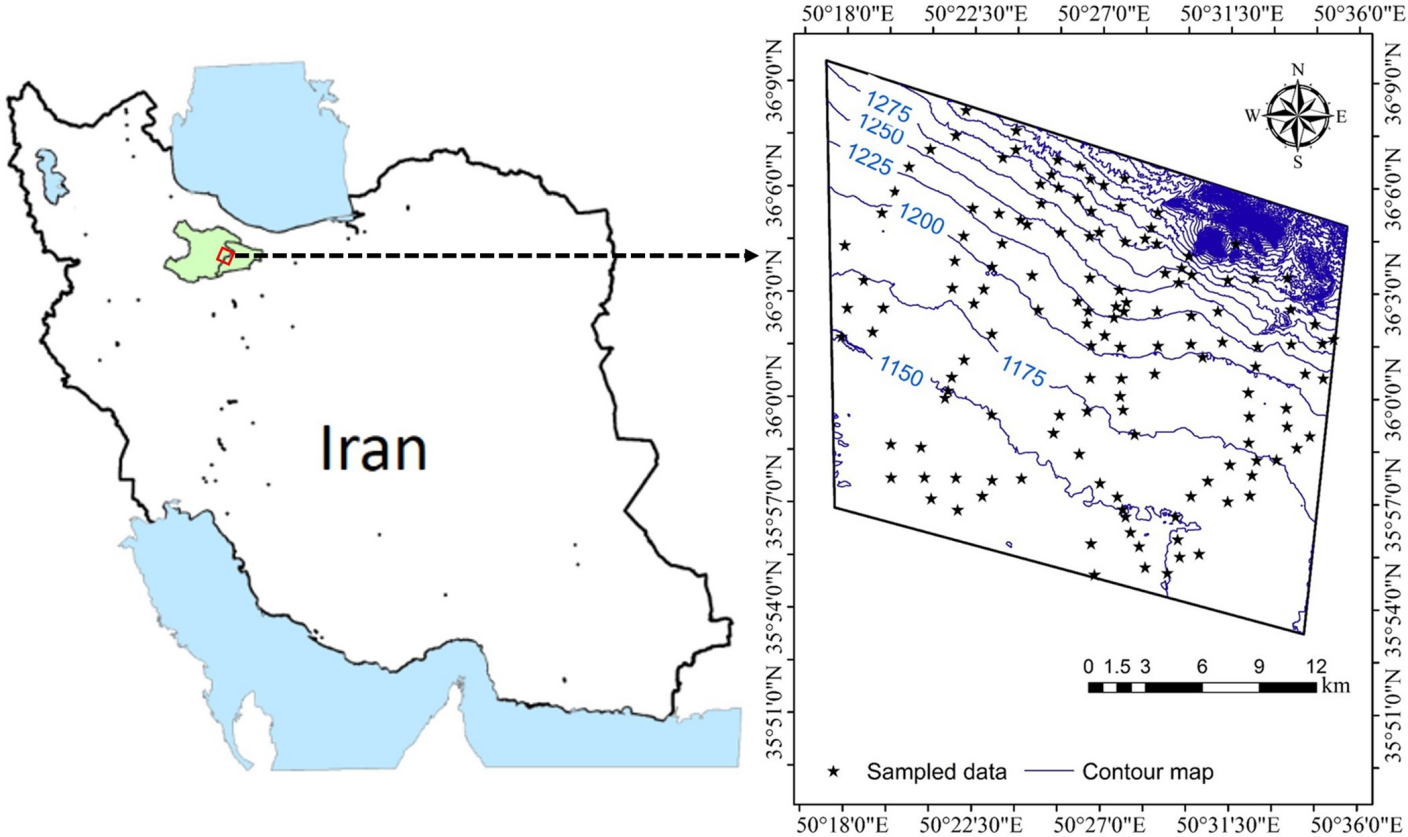
The studied area map and locations of sampled soils.

This study was conducted in the Qazvin province, situated in the central part of Iran. The study area encompasses geographic coordinates ranging from 35° 56.85’ to 36° 5.3’ latitude and 50° 21.3’ to 50° 33.9’ longitude, covering a total area of approximately 287 km^2^ (Fig 1). The topography of the region exhibits variations in elevation and slope, with elevations ranging from 1105 to 2423 meters above sea level and slopes ranging from 0.0% to 44.5%. To gain insights into the climatic conditions of the study area, long-term meteorological data collected by the Iran Meteorological Service from 2000 to 2022 were analyzed. The recorded data indicated an annual precipitation of 296 mm year^-1^, while the mean annual temperature was 14.5°C. These climatic conditions classify the prevailing climate in the study area as semi-arid, characterized by relatively low precipitation levels and moderate temperatures. The primary land use types observed in the central part of Iran, within the study area, include agricultural lands, range lands, and bare lands. Agricultural lands account for approximately 53% of the region, indicating a significant agricultural presence. The cultivation of crops such as alfalfa, winter wheat, and winter barley are prevalent in this agricultural landscape. Range lands, covering around 31% of the study area, provide grazing areas for livestock. Additionally, approximately 16% of the region consists of bare lands, which are devoid of vegetation cover.

### 2.2. Sampling and soil analysis

The soil sampling process in this study involved collecting samples from 150 points within the study area, specifically targeting a depth of 0–30 cm. To ensure representative sampling, a stratified random method was employed, taking into account topographical factors such as slope and elevation data. After collection, the soil samples were transported to the soil laboratory, where they were air dried to remove excess moisture. To determine soil salinity, saturated paste extracts were prepared from the soil samples. In this method, a 250 g portion of each soil sample was saturated with distilled water. The samples were then allowed to stand overnight to ensure complete saturation. Subsequently, the soil solution was extracted from the samples using suction filtration. The electrical conductivity (ECe) of the extracted solution was determined following the procedure described [15, 16]. In addition, soil texture fractions were also determined by hydrometer procedure, as outlined [17]. The calcium carbonate equivalent content was measured applying back-titration approach [18]. The soil organic carbon content was determined on the basis of wet-oxidation [19].

### 2.3. Spectral Vis-NIR measurements

The air-dried soil samples, which had been sieved to a particle size of less than 2mm, underwent further drying in a container with a 4 cm diameter. This process took place at a controlled temperature of 30°C for a duration of 10 hours. The purpose of this additional drying step was to ensure that the soil samples were uniformly dry and ready for spectral analysis. For obtaining the reflectance spectra of the soil samples, a portable spectrometer, specifically the FieldSpec 3 by Analytical Spectral Devices (ASD Inc.), was utilized. The measurements were conducted in a dark room to minimize external light interference. The spectrometer had a wide range of wavelengths recorded, spanning from 350 to 2500 nm. However, different resolutions were used for different wavelength ranges. From 350 to 1000 nm, a resolution of 1.4 nm was employed, while from 1000 to 2500 nm, a resolution of 2 nm was utilized. The spectrometer was equipped with a pistol grip, which facilitated the collection of reflectance spectra from each soil sample. To ensure accuracy and consistency in the reflectance measurements, a calibration process was followed. For every 10 soil samples, the recorded spectra were calibrated to reflectance values using a standardized white reference panel. This reference panel had a size of 15 by 15 cm and served as a baseline for obtaining accurate reflectance measurements. In order to reduce any potential noise in the reflectance data, the first and last sides of the recorded spectra were eliminated. Specifically, wavelengths ranging from 350 to 399 nm and from 2401 to 2500 nm were discarded. This step aimed to enhance the quality and reliability of the reflectance data, ensuring that any extraneous signals or disturbances at the edges of the spectrum were minimized.

### 2.4. Modeling strategy

#### 2.4.1. Multiple linear regression (MLR)

MLR is a widely employed approach for developing regression-based models to estimate unknown factors, such as soil properties [20–23]. The MLR model is typically represented by Eq (1):

y=Xβ+ε (1)

In this equation, y represents the vector of unknown factors with dimensions (n×1), X denotes the matrix with dimensions (n×p), ε indicates the residual vector with dimensions (n×1), and β represents the vector of regression coefficients with dimensions (p×1). The primary objective of developing regression models is to determine the optimal values of β by minimizing the sum of squared errors, as depicted in Eq (2):

$$\underset{\beta }{\min}∥y-x\beta {∥}_{2}^{2}$$ (2)

where, subscript 2 represents the L2-norm of the vector, which quantifies the error. The derivation of the MLR model assumes that the residuals follow a normal distribution. Additionally, several assumptions are made, including E(ε) = 0 (residuals have a mean of 0) and Var(ε) = σ^2^ (residuals have constant variance). Furthermore, it is assumed that the spatial distribution of residuals is independent and random, meaning that the residual at one point i (ε_*i*_)) is not correlated with the residual at the next point (ε_*i*+1_). To assess the validity of these assumptions, various statistical tests were employed in this study. The histogram of the residuals was examined to evaluate their distribution and assess the normality assumption. Additionally, the Kolmogorov–Smirnov (K-S test) was utilized to statistically test the normal distribution of the residuals. Another important consideration in MLR analysis is the presence of multi-collinearity, which occurs when the predictor variables are highly correlated. To detect multi-collinearity, the variance inflation factors (VIFs test) were employed. The VIF measures the extent to which the variance of the estimated regression coefficient is inflated due to multi-collinearity [24, 25].

#### 2.4.2. Random forest (RF)

Random forest is a powerful machine learning method that leverages the principles of classification and regression trees (CART) along with ensemble learning techniques. This method, initially introduced by Breiman in 2001, has gained significant popularity and proven to be highly effective in various applications. At its core, random forests combine the predictions of multiple individual decision trees to obtain a robust and accurate model. Each decision tree within the random forest is constructed using a random subset of the training data and a subset of the input features, thereby introducing randomness and diversity into the model. This diversity, coupled with the aggregation of predictions from multiple trees, helps to mitigate overfitting and improve generalization performance.

The process of building a random forest involves creating an ensemble of decision trees through a bootstrap sampling technique. The training data is randomly resampled with replacement to generate multiple subsets, each of which is used to build an individual decision tree. Additionally, for each tree, a subset of input features is randomly selected, limiting the number of features considered during the tree construction process. This random feature selection further enhances the diversity among the trees and promotes robustness. During the prediction phase, each tree in the random forest independently generates a prediction based on the input features. For classification tasks, the final prediction is determined through majority voting, where the class with the most votes across the ensemble is selected. In regression tasks, the predictions of individual trees are averaged to obtain the final output. The key advantages of random forests lie in their ability to handle high-dimensional data, identify important input features, and handle missing values effectively. Furthermore, random forests offer built-in mechanisms for assessing feature importance, enabling insights into the relative contributions of different features in the model’s predictions.

#### 2.4.3. Input variables to develop different types of models

In this study, the derivation of models was performed considering three scenarios for each land use category and the entire dataset. The details of these scenarios are presented in Table 1.

**Table 1:** The input variables for deriving different types of models are used to predict soil salinity.

Scenarios	Spectral reflectance wavelength	Combining spectral reflectance wavelength
Scenario I	+	-
Scenario II	-	+
Scenario III	+	+

+ shows variables as inputs to predict soil salinity, while ‐ means not.

### 2.5. Performance criteria

To evaluate the performance of the derived regression models, a careful division of the soil samples was conducted for both training and testing purposes. In this study, a random split was performed, allocating 20% of the samples to the testing data-set and the remaining 80% to the training data-set. To assess the performance of the developed models under various scenarios, several well-established evaluation criteria were employed. These criteria included the ratio of performance deviation (RPD), coefficient of determination (R^2^), and root mean square error (RMSE). RPD is a measure of the ratio between the standard deviation of the reference values and the root mean square error, providing insight into the quality of the predictions. R^2^, on the other hand, quantifies the proportion of the variance in the dependent variable that can be explained by the regression model. Lastly, RMSE represents the square root of the average squared differences between the predicted and observed values, serving as a measure of the model’s accuracy [17, 22, 24, 25].

$$\text{RMSE}={\left[\frac{\sum_{\text{i=1}}^{\text{N}}{\left({\hat{Y}}_{1}-{\text{Y}}_{\text{i}}\right)}^{2}}{\text{N}}\right]}^{0.5}$$ (3)

$${\text{R}}^{2}=1-\frac{\sum_{\text{i=1}}^{\text{N}}{\left({\text{Y}}_{\text{i}}-{\hat{Y}}_{1}\right)}^{2}}{\sum_{\text{i=1}}^{\text{N}}\left(Y_{i}^{2}\right)-\frac{{\left(\sum_{\text{i=1}}^{\text{N}}{\text{Y}}_{i}\right)}^{2}}{N}}$$ (4)

$$RPD=\frac{SD}{RMSE}$$ (5)

which Y_i_ represents the measured data obtained from the soil samples, ${\hat{Y}}_{1}$ represents the estimated data generated through models and N represents the number of soil samples included in the analysis. Moreover, the SD represents the characterizes the variability or dispersion of the measured ECe values.

## 3. Results and discussions

### 3.1. Statistical analysis of soil characteristics

**Fig 2:**
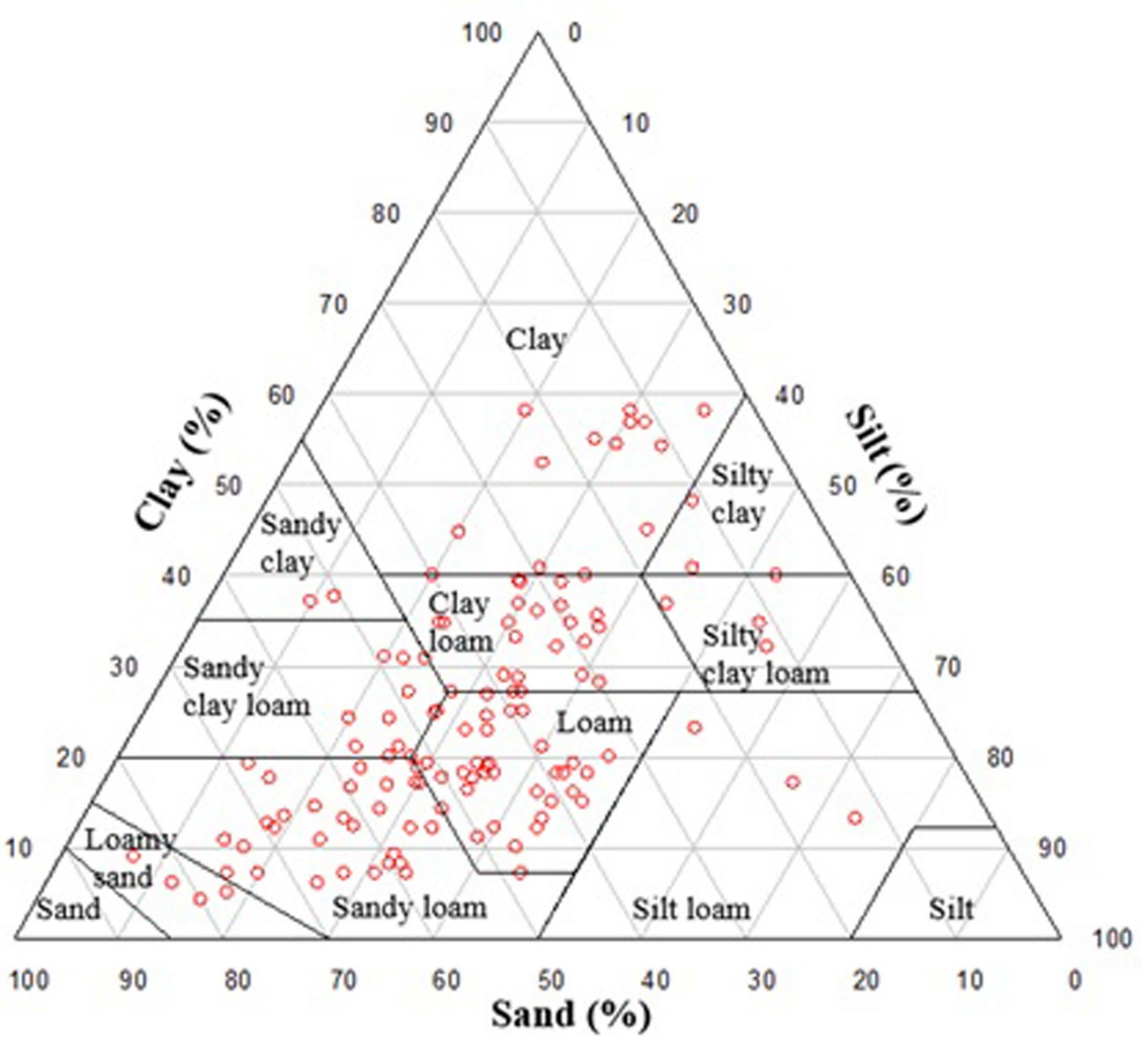
Soil texture classes of sampled soils according to the USDA classification.

**Fig 3:**
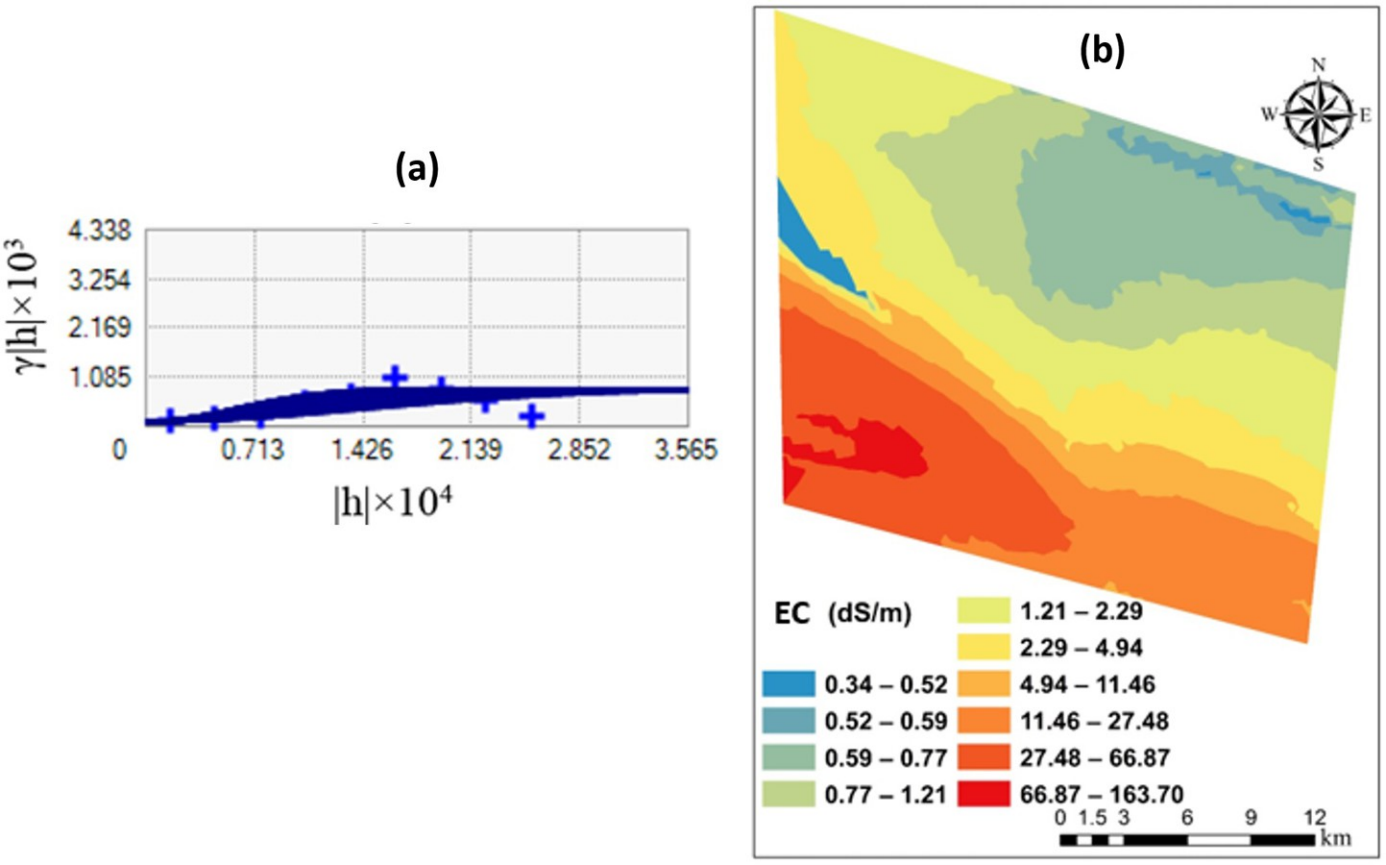
Soil salinity in the studied area: variogram (a) and map (b).

The mean percentages of silt, sand, and clay in the examined region were 27.0%, 40.8%, and 32.2%, respectively (Table 2). Based on the soil texture classification system (US Soil Taxonomy, USDA, 2010), the prevalent soil texture classes included loam, silt loam, sandy loam, and clay loam (Fig 2). The calcium carbonate equivalent values for the studied area ranged from 3.0 to 44.9%, with a mean value of 18.6% (Table 2). The EC_e_ measured data varied from 0.34 to 163.70 dS m^-1^, with an average of 15.43 dS m^-1^ (Table 2). As per the Kolmogorov-Smirnov test [26], the soil salinity data demonstrated a normal distribution with a significance level of p = 0.05. The variogram and map of soil salinity in this region are presented in Fig 3. The soil salinity in about 41% of this region is under 4 dS m^-1^, indicating that the soil is within the normal range for most crops (Fig 3).

**Table 2:** The descriptive statistics for soil properties.

Soil property	Min	Max	Mean
Clay (%)	5.0	56.0	32.2
Sand (%)	4.0	86.0	40.8
Silt (%)	8.0	76.0	27.0
EC (dS m^-1^)	0.34	163.70	15.43
SOC (%)	0.24	1.03	0.65
CCE (%)	3.0	44.9	18.6

EC: Electrical conductivity; SOC: Soil organic carbon; CCE: Calcium carbonate equivalent.

Table 3 provides a detailed summary of the observed soil salinity data in farmlands, rangelands, and bare lands. The soil salinity data indicate notable variations within the dataset, spanning from 0.9 to 48.0 dS m^-1^ for farmlands, 0.9 to 34.8 dS m^-1^ for rangelands, and 1.2 to 140.0 dS m^-1^ for bare lands (Table 3). The slopes of bare lands are predominantly nearly flat, and they indicate a significantly higher mean soil salinity due to the proximity of the water table to the soil surface. Developing a reliable predictive model for soil salinity across different land use types could be highly significant. A crucial aspect of modeling is the need for a diverse and extensive data set.

**Table 3:** The properties of EC (dS m^-1^) measured value at different land use types.

Land use	Min	Max	Mean
Farmlands	0.9	48.0	5.9
Rangelands	0.9	34.8	5.4
Bare lands	1.2	140.0	47.1

### 3.2. Spectral reflectance signatures of different types of land use

**Fig 4:**
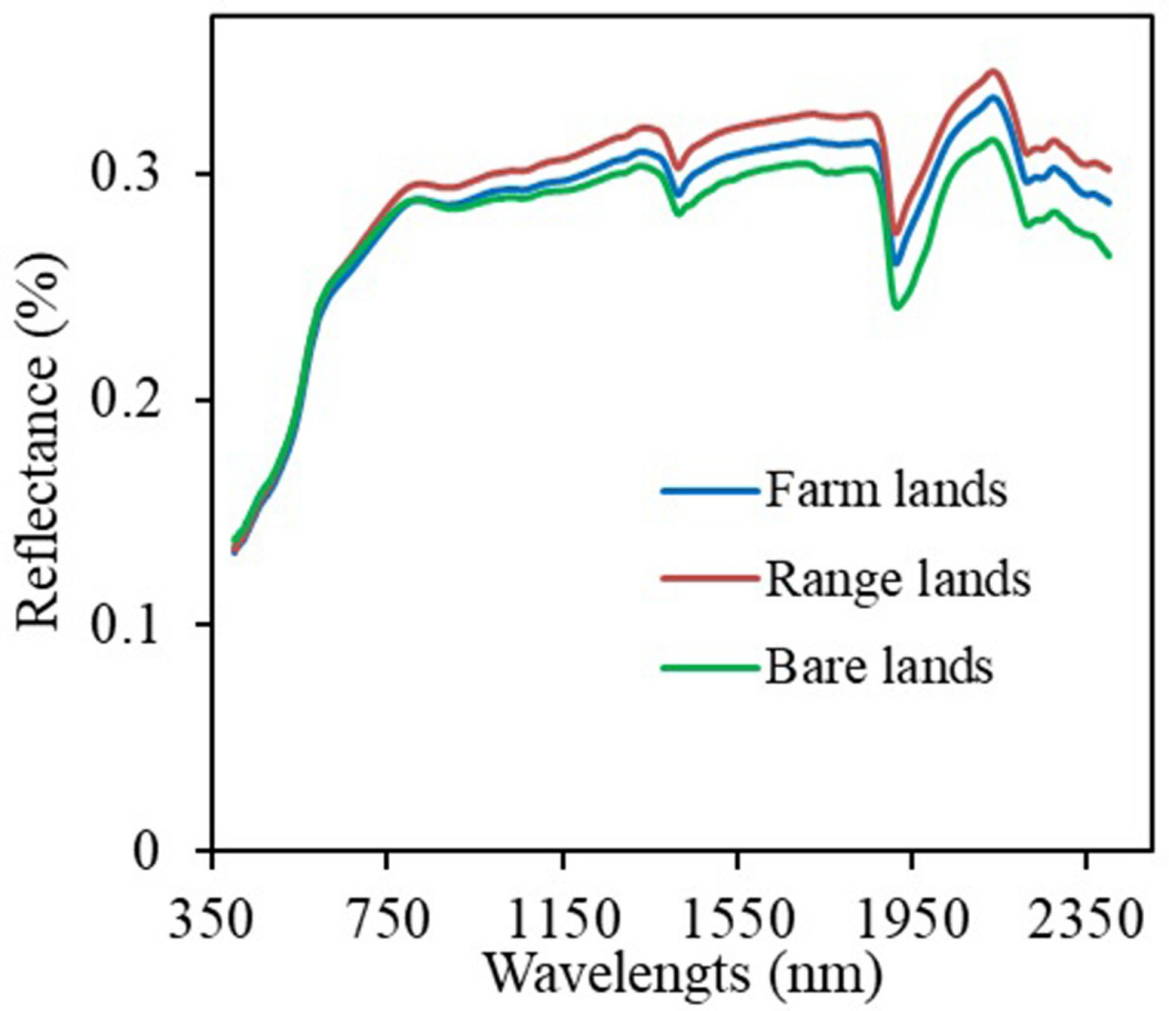
Changes in spectral reflectance for different types of land use.

The spectral signatures of soils affected by salts under different land use types are presented in Fig 4. As seen in Fig 4, the lowest and highest mean reflectance were observed for bare lands and farmlands, respectively. The lowest reflectance in bare lands soils could be attributed to the higher level of organic carbon at the soil surface [24]. In bare lands, dissolved humus migrates to the soil surface through capillary water flow, resulting in a thin black layer at the soil surface [25]. Several research [27–29] have found that soil salinity at the soil surface increases reflectance in different bands such as blue, green, red and NIR.

### 3.3. Deriving predictive models by using whole soil salinity measured data

The predictive models generated from the entire soil salinity dataset, such as MLR and RF, are present in Table 4. In the ongoing study, specific models, as outlined in Table 4, were formulated by considering the correlation coefficient values between soil salinity and spectral reflectance at various wavelengths. These models aim to capture the interplay and relationships existing between ECe and the spectral characteristics across different wavelength ranges. Considering these correlations contributes to a more nuanced understanding and accurate prediction within the research framework.

**Table 4:** Derived predictive models to predict soil salinity by using whole measured data.

Predictive models		Train		Test		
		RMSE (dS m^-1^)	R^2^	RMSE (dS m^-1^)	R^2^	RPD
**MLR**						
*Scenario I*	$EC_{e}=59.28-150.43R_{1980}$	12.24	0.34	14.09	0.28	1.32
*Scenario II*	$EC_{e}=396.54-419.98\left(\frac{R_{1980}}{R_{1448}}\right)$	8.54	0.48	8.31	0.50	1.43
*Scenario III*	$EC_{e}=120.89-443.62R_{1980}+44.26\left(\frac{R_{1980}}{R_{1448}}\right)$	7.10	0.68	7.29	0.64	1.85
**RF**						
*Scenario I*	-	8.02	0.52	8.18	0.49	1.68
*Scenario II*	-	5.67	0.81	5.45	0.78	2.46
*Scenario III*	-	4.89	0.86	4.85	0.87	3.15

The results of derived predictive models for different types of datasets, such as training and testing, are shown in Table 4. In this study, spectral reflectance data in 1980 nm and the ratio of 1980 to 1448 nm as a combining factor had the highest correlation with soil salinity data. For this reason, these factors were used as scenarios I, II, and III (Table 4). Table 4 shows that using combining spectral data performed better than using spectral data alone. To enhance the predictive models, the input factors in scenarios I and II were used in Scenario III. The results in Table 4 showed that integrating input variables from scenario I and II increased the model performance. Generally, the RF model performed better result than the MLR model, considering statistical indices presented in Table 4. Some statistical indices, such as RPD and R^2^, grouped the predative model performance as poor if RPD<1.5 and R^2^<0.60, moderate if RPD from 1.5 to 2 and R^2^≥ 0.60, good if RPD from 2 to 2.5 and R^2^≥0.70, and excellent if RPD ≥2.5 and R^2^≥ 0.80 [30, 31].

As indicated by the findings presented in Table 4, the predictive MLR model, when using combining factors in scenario III, showed a moderate performance in predicting soil salinity in studied area. From Table 4, it can be seen that the RF model indicated excellent performance by using combined factors in scenario III. Consistent with these results, previous studies [11, 14] have highlighted the utilization of spectral data in similar prediction tasks. One study focused on monitoring saline soils within the Ebinur Lake Wetland National Nature Reserve in central Asia. By employing Vis-NIR spectra data, it successfully assessed the spatial distribution and temporal changes of soil salinity. This research not only emphasized the effectiveness of Vis-NIR spectroscopy in salinity monitoring but also highlighted its potential to inform management strategies within environmentally sensitive areas [11]. Vis-NIR spectra were also applied in a study using a partial least squares regression (PLSR) model to predict soil salinity on the eastern coast of the Urmia hypersaline lake in Iran. Their study demonstrated the utility of Vis-NIR spectroscopy combined with advanced statistical techniques in accurately estimating soil salinity levels, thus providing valuable information for agricultural planning and land management in the region [14]. However, Thabit et al. [32] showed the RF forecasting models have low accuracy in prediction of soil properties such as soil organic matter.

### 3.4. Deriving predictive models for different types of land use

The results of the developed models for the best scenarios under different types of land use are presented in Table 5. The best scenario for farmlands, rangelands, and bare lands were scenario III, III, and I, respectively. According to Table 5, the derived regression model in this study described for 25, 36, and 62% of the soil salinity variations under farmlands, rangelands, and bare lands, respectively (Table 5). However, the derived RF model performed better than the regression model across various types of land use (Table 5). The statistical indices, such as RPD and RMSE for bare lands, rangelands, and farmlands, were 1.61 and 1.49, 3.19 and 1.10, and 3.62 and 16.51, respectively (Table 5). The RF model, as the best model in the current study, explained 82, 65, and 46% of the soil salinity variations under bare lands, rangelands, and farmlands, respectively (Table 5). The poor performance of the derived models under farmlands could be attributed to the complicated nature of farmlands, that are disturbed annually in this region. Additionally, soil under range lands in some parts of the studied area are destroyed because of overgrazing. Moreover, in bare lands, the absence of vegetation may lead to increased exposure of the soil surface to factors like evaporation and capillary rise, which can contribute to the concentration of salts at the surface [11, 14, 33]. For this reason, bare lands in the studied region had higher salts on the soil surface and consequently, salt concentration can affect the soil reflectance [34–36]. Finally, the outcomes of this study underscore the effectiveness of utilizing combined spectral reflectance from various wavelengths in predicting soil salinity for some land use types.

**Table 5:** Derived predictive models for estimating soil salinity under different land use types.

Predictive models		Train		Test		
		RMSE (dS m^-1^)	R^2^	RMSE (dS m^-1^)	R^2^	RPD
***Farmlands***						
MLR	$EC_{e}=158.73-169.32R_{1980}+8.71\left(\frac{R_{1980}}{R_{1448}}\right)$	2.42	0.27	2.59	0.25	1.119
RF	—	1.35	0.49	1.49	0.46	1.61
***Range lands***						
MLR		$EC_{e}=44.61-42.71R_{1980}+22.14\left(\frac{R_{1980}}{R_{1448}}\right)$	1.84	0.37	1.80	0.36	1.45
RF	—	1.07	0.68	1.10	0.65	3.19
***Bare lands***						
MLR	*** $EC_{e}=119.82-269.73R_{1980}$ ***	24.39	0.56	22.09	0.62	3.00
RF	—	15.91	0.88	16.51	0.82	3.62

## 4. Conclusion

In this research, the objective was to develop different types of models to accurately predict soil salinity. To achieve this, the Vis-NIR spectroscopy data were applied. The results revealed that the derived models, incorporating spectral reflectance data consistently outperformed across all datasets, farmlands, and rangelands. However, more analysis showed that RF model was the best model under various types of land use. The RF model can explain 82, 65, and 46% of soil salinity variations under bare lands, rangelands, and farmlands, respectively. These findings highlight the considerable improvement in prediction accuracy by combining spectral reflectance data and suggest a method to explore soil salinity prediction for future research in various topographical conditions.

## Supporting information

S1 Data (XLSX)
